# Molecular epidemiology of *mcr-1*-harboring avian pathogenic *Escherichia coli* from diseased chickens in Shanxi, China (2021–2024)

**DOI:** 10.3389/fmicb.2026.1883400

**Published:** 2026-08-11

**Authors:** Li Fangfang, Wang Haiyang, Shan Pengju, Wang Jingying, Hu Jiangang, Lian Liyan, Wang Zhihao, Saqib Nawaz, Bao Yinli, Han Xiangan, Wang Haidong

**Affiliations:** 1 College of Veterinary Medicine, Shanxi Agricultural University, Taigu, China; 2 The Chinese Academy of Agricultural Sciences (CAAS), Shanghai Veterinary Research Institute, Shanghai, China; 3 Key Laboratory of Fujian Universities Preventive Veterinary Medicine and Biotechnology, Longyan University, Longyan, China

**Keywords:** avian pathogenic *Escherichia coli*, biofilm formation, MCR-1, molecular epidemiology, multidrug resistance

## Abstract

**Introduction:**

The emergence of mcr-1-harboring avian pathogenic *Escherichia coli* (APEC) poses a significant One Health challenge due to its zoonotic potential and adverse effects on poultry production.

**Methods:**

A total of 50 *mcr-1*-positive APEC isolates were recovered from diseased chickens in Shanxi Province, China, between 2021 and 2024. All isolates were subjected to serotyping, phylogenetic grouping, antimicrobial susceptibility testing, detection of resistance and virulence-associated genes, and biofilm formation assessment.

**Results:**

Serotyping identified O18 (28%) and O78 (20%) as the predominant serotypes, with no O1 detected. Phylogenetic analysis showed that phylogroup A was most prevalent (32.0%), followed by B2 (12.0%), D (12.0%), and B1 (10.0%); the remaining isolates (34.0%) were untypeable. All isolates were multidrug-resistant (resistant to ≥3 drug classes), with universal resistance to tetracycline and trimethoprim-sulfamethoxazole, and over 85% resistance to kanamycin, amoxicillin, florfenicol, and chloramphenicol. The resistance genes *tetA*, *blaTEM*, *aphA*, and *sul2*, as well as the virulence genes *ibeB* and *ompA*, were detected in all isolates (100%). Biofilm formation was observed in 96% of the isolates, though its correlation with individual resistance or virulence genes was weak.

**Discussion:**

The findings demonstrate that *mcr-1*-positive APEC isolates in Shanxi Province exhibit high-level multidrug resistance, widespread virulence gene carriage, and strong biofilm-forming capacity, underscoring their potential public health risk through animal-to-human transmission. These data provide a baseline for local surveillance and highlight the need for enhanced antimicrobial stewardship in poultry production.)

## 1 Introduction

Avian pathogenic *Escherichia* coli (APEC) is an extraintestinal pathogenic *E. coli* (ExPEC) with a broad host range (Fu et al., 2023). Avian colibacillosis caused by APEC has persisted in poultry production for more than 50 years and remains one of the most important bacterial diseases in the industry (Johnson et al., 2022). This disease often leads to localized or systemic infections in poultry, including respiratory infections, septicemia, polyserositis, and cellulitis, resulting in substantial economic losses to the poultry industry (Yin et al., 2022). In addition, APEC poses a potential public health risk to humans, as some APEC strains are highly homologous to human ExPEC, share multiple virulence factors, exhibit close phylogenetic relationships, and may cause disease in mammals (Jamali et al., 2024; Wang et al., 2022). Moreover, most APEC strains harbor ColV or ColV-like plasmids that encode multiple virulence genes and have been shown to contribute to their pathogenicity in poultry (Habouria et al., 2019).

Antimicrobial resistance (AMR), particularly multidrug resistance (MDR) to multiple antibiotics, is recognized as one of the greatest global public health threats of the 21st century (Zhao et al., 2022). The large-scale and indiscriminate use of antibiotics in veterinary practice has contributed to the increasing prevalence of multidrug-resistant APEC strains (Hai et al., 2025; Seiffert et al., 2013). Moreover, *E. coli* can acquire AMR genes through various mobile genetic elements (MGEs), and these resistance determinants can further disseminate among humans, animals, and the environment via horizontal gene transfer (HGT) (Shoaib et al., 2025). Polymyxins are generally regarded as last-resort antibiotics for the treatment of infections caused by Gram-negative bacteria; however, their clinical effectiveness is increasingly compromised by the global spread of plasmid-mediated colistin resistance genes (*mcr*) (Nawaz et al., 2024; Nang et al., 2019). To date, ten mcr variants have been identified, among which *mcr-1* is the most prevalent and the earliest discovered (Ramaloko and Osei Sekyere, 2022). The rapid evolution and dissemination of the mcr gene family pose a serious threat to global public health (Liang et al., 2025).

As one of the major virulence factors of APEC, biofilm formation enhances resistance to host immune defenses and improves bacterial survival in the environment (Hu et al., 2022). Biofilms are complex microbial communities characterized by a dynamic, adhesive, and protective extracellular matrix composed mainly of polysaccharides, proteins, and nucleic acids (Carradori et al., 2020). The presence of biofilms surrounding bacterial cells can partially or completely reduce the efficacy of antimicrobial agents, thereby increasing antibiotic tolerance and further contributing to the emergence and spread of AMR (Idrees et al., 2021). Therefore, epidemiological investigations supported by molecular approaches that assess antimicrobial resistance profiles, virulence-associated genes, resistance determinants, and biofilm-forming capacity of APEC are essential for the prevention and control of *E. coli* infections in poultry farms (Quaresma et al., 2022; Li et al., 2021).

This study aimed to systematically characterize the molecular epidemiological features of *mcr-1*-positive isolates obtained from 356 chickens suspected of colibacillosis in Shanxi Province, China, between 2021 and 2024, and to analyze the associations between biofilm-forming capacity and multiple related factors. These molecular epidemiological investigations are of great importance for controlling the emergence of multidrug-resistant APEC strains and for reducing the risk of antimicrobial resistance transmission from poultry to humans through the food chain.

## 2 Materials and methods

### 2.1 Isolation and identification of *mcr-1*-positive APEC

Between 2021 and 2024, a total of 356 chickens suspected of colibacillosis were collected from broiler farms in Shanxi Province, China. Samples were streaked onto eosin methylene blue (EMB) agar (Oxoid Company, Manchester, United Kingdom), and single colonies exhibiting a metallic green sheen were selected and inoculated into Luria–Bertani (LB) broth (Qingdao Haibo Biotechnology, China), followed by incubation at 37 °C overnight. Bacterial genomic DNA was extracted using a commercial genomic DNA extraction kit (Tiangen Biotech, China) according to the manufacturer’s instructions. The isolates were confirmed as *E. coli* by PCR amplification of the *phoA* gene using primers *phoA-F/phoA-R* (F: CGATTCTGGAAATGGCAAAAG; R: CGTGATCAGCGGTGACTATGAC), with a PCR reaction lacking template DNA included as a negative control. From the 356 samples, a total of 289 *E. coli* isolates were recovered. All 289 isolates were subsequently screened for the *mcr-1* gene by PCR using primers mcr-1-F/mcr-1-R (F: CGGTCAGTCCGTTTGTTC; R: CTTGGTCGGTCTGTAGGG), yielding 50 *mcr-1*-positive strains (17.30% positivity rate among *E. coli* isolates). To ensure epidemiological independence, only one isolate per chickens was retained for further analysis. Bacterial genomic DNA was extracted using a commercial genomic DNA extraction kit (Tiangen Biotech, China) according to the manufacturer’s instructions. Detailed sampling site distribution and isolation rates are presented in Supplementary Table S1.

### 2.2 Serotyping analysis

Serotyping of APEC was performed according to the method described by Fancher et al. (2021). Serotype-specific primers (Supplementary Table S2) were used for PCR amplification with genomic DNA from the isolates as templates. Each PCR reaction mixture (20 μL) contained 10 μL of 2 × Es Taq MasterMix Dye, 7 μL of ddH_2_O, 1 μL of each forward and reverse primer, and 1 μL of DNA template. The PCR cycling conditions were as follows: initial denaturation at 98 °C for 1 min; 35 cycles of denaturation at 98 °C for 15 s, annealing at 55 °C for 15 s, and extension at 72 °C for 15 s; followed by a final extension at 72 °C for 10 min. All PCR products were analyzed by electrophoresis on 1.0% agarose gels (Sangon Biotech, China) to determine amplicon sizes, and the amplification results were recorded.

### 2.3 Phylogenetic grouping analysis

Phylogenetic grouping of *E. coli* isolates was determined according to the method described by Clermont et al. (2000), based on the presence or absence of the *chuA* and *yjaA* genes and the *TspE4. C2* DNA fragment. PCR assays targeting these genetic markers were performed using genomic DNA from the isolates as templates, with primer sequences listed in Supplementary Table S3. Each PCR reaction (20 μL) consisted of 10 μL of 2 × Es Taq MasterMix Dye, 7 μL of ddH_2_O, 1 μL of each forward and reverse primer, and 1 μL of DNA template. The PCR cycling conditions were as follows: initial denaturation at 94 °C for 4 min; 30 cycles of denaturation at 94 °C for 4 min, annealing at 50–60 °C for 40 s, and extension at 72 °C for 1 min; followed by a final extension at 72 °C for 10 min. PCR products were analyzed by electrophoresis on 1.0% agarose gels, and amplicons of the expected size were purified and sequenced by Sangon Biotech. Sequence identity was confirmed by BLAST analysis (BLAST: Basic Local Alignment Search Tool).

### 2.4 Antimicrobial susceptibility testing

Antimicrobial susceptibility testing was performed using the disk diffusion (Kirby–Bauer) method according to the Clinical and Laboratory Standards Institute (CLSI) guidelines (M100, 32nd edition, 2022). *Escherichia coli* ATCC 25922 was used as the quality control strain, with inhibition zone diameters verified to fall within the expected ranges specified by CLSI (Feng et al., 2023). Bacterial suspensions (approximately 1 × 10^7^ CFU/mL) were evenly spread onto Mueller–Hinton (MH) agar (AOBOX Biotech, China) plates. After complete absorption, antimicrobial disks (Microbial Reagent Co., China) containing chloramphenicol (CAP, 30 μg), florfenicol (FFC, 30 μg), amoxicillin (AMX, 20 μg), ceftazidime (CAZ, 30 μg), enrofloxacin (ENFX, 10 μg), ofloxacin (OFX, 5 μg), tetracycline (TC, 30 μg), trimethoprim–sulfamethoxazole (SMZ-TMP, 19 μg), amikacin (AMK, 30 μg), and kanamycin (KAN, 30 μg) were placed on the agar surface using sterile forceps. Plates were incubated at 37 °C for 18 h, after which the diameters of inhibition zones were measured and recorded. Interpretation of susceptibility (susceptible, intermediate, or resistant) was based on the CLSI breakpoints for each antibiotic. All assays were performed in triplicate, and the mean inhibition zone diameters were used for subsequent analysis.

### 2.5 Detection of antimicrobial resistance genes

PCR assays were performed to detect five common classes of antimicrobial resistance genes in *E. coli* (Ngai et al., 2021), including tetracycline resistance genes (*tetA*), amphenicol resistance genes (*cat1* and *floR*), *β*-lactam resistance genes (*blaTEM* and *blaCTX-M*), aminoglycoside resistance genes (*strA* and *aphA*), and sulfonamide resistance genes (*sul1* and *sul2*). The primer sequences used for amplification are listed in Supplementary Table S4. The PCR reaction mixtures and cycling conditions were performed as described above.

### 2.6 Detection of virulence-associated genes

PCR assays were conducted to detect common virulence-associated genes in *E. coli*, including adhesin-related genes (*aatA, papC, tsh, fimC, mat,* and *vat*), iron acquisition–related genes (*fyuA, iucD,* and *irp2*), invasion-associated genes (*ibeB, yijP*, and *ibeA*), and serum resistance–associated genes (*ompA, neuC, cva, iss*, and *iroN*) (Kathayat et al., 2021; Thomrongsuwannakij et al., 2020). Gene-specific primers were designed for amplification, and the primer sequences are listed in Supplementary Table S5. Genomic DNA from the isolates was used as the template, and the PCR reaction mixtures and cycling conditions were performed as described above.

### 2.7 Biofilm formation assay

Biofilm formation by the isolates was assessed using the crystal violet staining method (Yin et al., 2022). Single colonies of APEC isolates were inoculated into tubes, and bacterial suspensions adjusted to an OD_600_ of 1.0 were diluted 1:100 with LB broth and dispensed into sterile 96-well microtiter plates (200 μL per well), with six replicate wells for each isolate. Plates were incubated statically at 37 °C for 24 h, with sterile LB broth and 1% lactose-supplemented LB broth included as blank controls. After incubation, the culture medium was discarded, and the wells were gently washed twice with phosphate-buffered saline (PBS). Following air drying, 200 μL of 0.1% (w/v) crystal violet (Coolaber, China) solution was added to each well and incubated at 37 °C for 30 min. The crystal violet solution was then removed, and the wells were washed twice with PBS, air-dried at room temperature, and treated with 200 μL of 95% ethanol to solubilize the bound dye at 37 °C for 10 min. The absorbance was measured at 595 nm using a microplate reader (ThermoFisher, United States), and the results were recorded and analyzed. Biofilm-forming ability was classified according to the criteria described by Li et al. (2021).

### 2.8 Statistical analysis

All experiments were independently repeated at least three times. Data are presented as the mean ± standard deviation (SD). Differences between groups were assessed using one-way analysis of variance (ANOVA). The linear correlation between two variables was evaluated with Pearson’s correlation coefficient (r). For invariant genes, the values were directly assigned as 0. For each gene–biofilm combination, the correlation coefficient was calculated, and the 95% confidence interval (CI) of r was estimated via nonparametric bootstrap resampling (1,000 iterations). To control the false positive risk arising from multiple comparisons, *p*-values were adjusted using the Benjamini–Hochberg false discovery rate (FDR) method. An adjusted *p* < 0.05 was considered statistically significant. Specifically, correlation strength was interpreted as follows: 0.5 ≤ |*r*| < 1.0, strong correlation; 0.3 ≤ |*r*| < 0.5, moderate correlation; 0.1 ≤ |*r*| < 0.3, weak correlation; and |*r*| < 0.1, no correlation. All statistical analyses were performed using GraphPad Prism (version 9.0) and R (version 4.5.2).

## 3 Results

### 3.1 Serotyping and phylogenetic analysis of mcr-1-positive isolates

**Figure 1:**
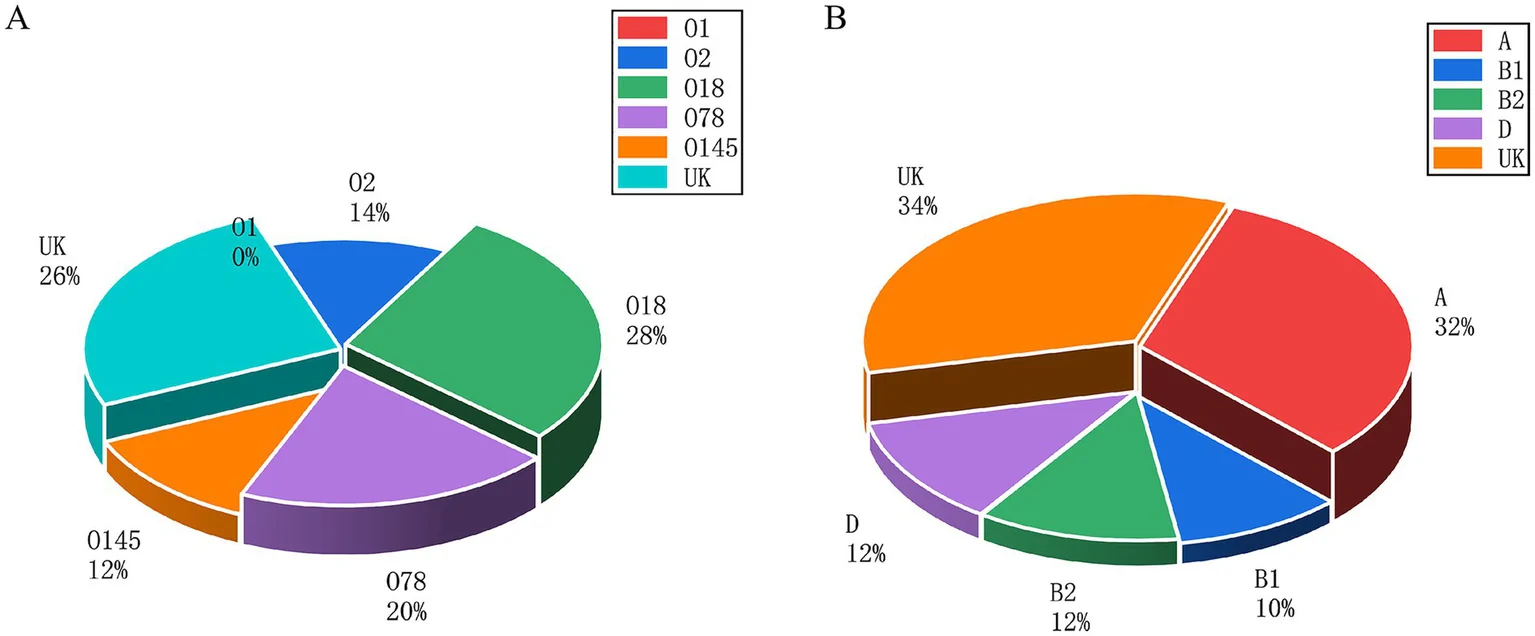
Serotype distribution and phylogenetic grouping of *mcr-1*-positive bacterial strains. **(A)** Red, dark blue, green, purple, orange, and light blue represent serogroups O1, O2, O18, O78, O145, and isolates not assigned to any of the tested serogroups, respectively. **(B)** Red, dark blue, green, purple, and orange indicate phylogenetic groups A, B1, B2, D, and unassigned groups, respectively. The proportion of each group is represented by the size and height of the pie segments.

PCR-based serotyping was performed to identify the O1, O2, O18, O78, and O145 serogroups among 50 *mcr-1*-positive isolates. The results showed that serogroup O18 was the most prevalent, with 14 isolates (28%), followed by O78 with 10 isolates (20%). Six isolates (12%) belonged to serogroup O145, and seven isolates (14%) were classified as O2. No isolates of serogroup O1 were detected. Among the 50 *mcr-1*-positive APEC isolates, 13 isolates (26%) could not be assigned to any of the tested serogroups (Figure 1A). To determine the phylogenetic distribution of the isolates, the presence of *chuA, yjaA*, and *TspE4. C2* was examined. As shown in Figure 1B, phylogroup A was the most common, accounting for 32.0% (16/50) of the isolates. In contrast, phylogroups B1, B2, and D were less frequent, representing 10% (5/50), 12% (6/50), and 12% (6/50), respectively. In addition, 34% (17/50) of the *mcr-1*-positive isolates could not be assigned to the established *E. coli* phylogenetic group.

### 3.2 Antimicrobial resistance phenotypes and multidrug resistance profiles

**Figure 2:**
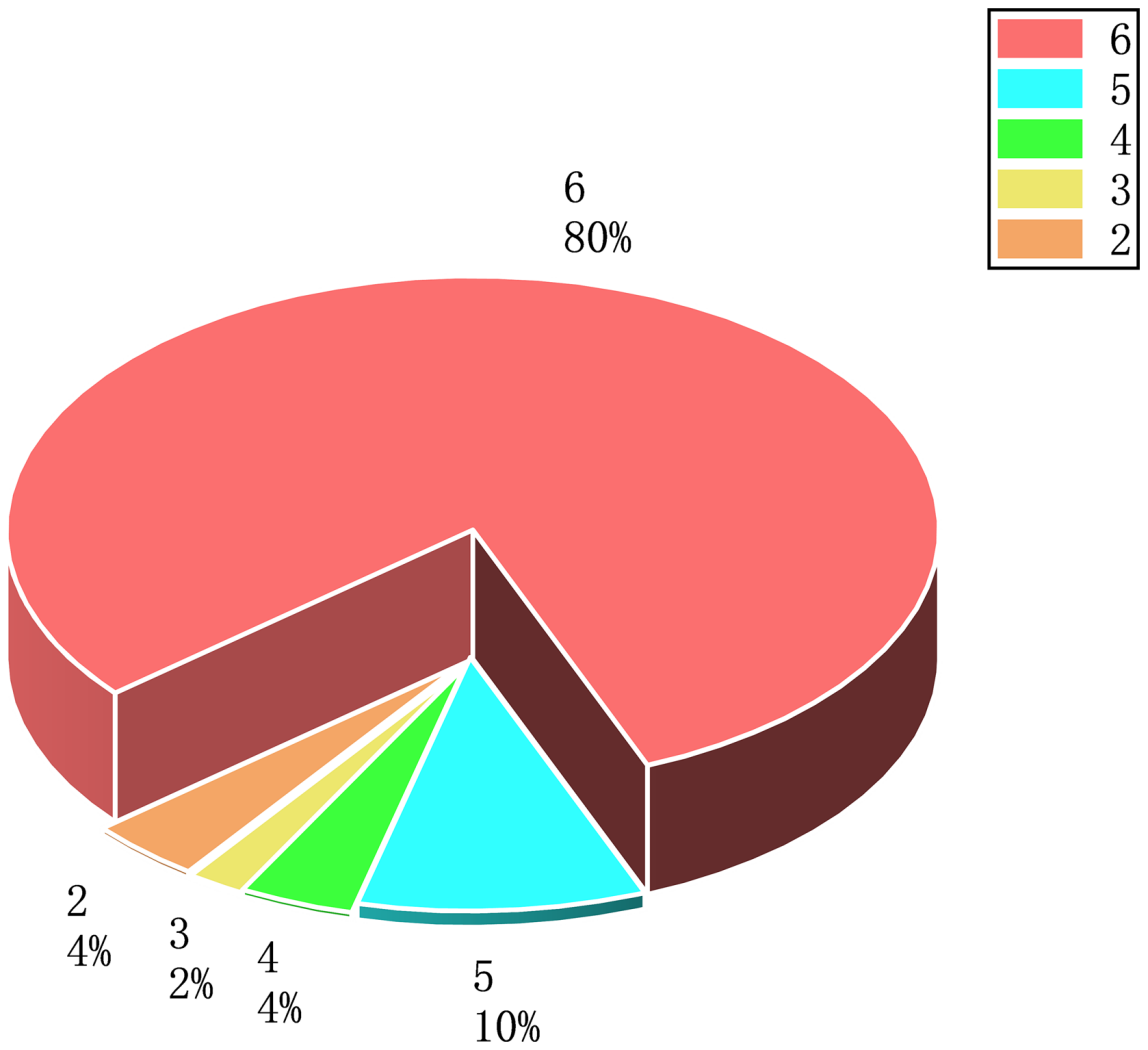
Prevalence of multidrug resistance among *mcr-1*-positive strains. Strains were grouped according to the number of antibiotic classes to which they were resistant. Red, blue, green, yellow, and orange represent resistance to six, five, four, three, and two antibiotic classes, respectively. The proportion of each group is visualized by the size and height of the pie segments.

Antimicrobial susceptibility testing showed that the 50 APEC isolates exhibited varying degrees of resistance to six classes of antibiotics, including amphenicols, *β*-lactams, quinolones, tetracyclines, sulfonamides, and aminoglycosides. All isolates were resistant to tetracycline and trimethoprim-sulfamethoxazole. High resistance rates were also observed for kanamycin (88%, 44/50), amoxicillin (94%, 47/50), florfenicol (88%, 44/50), and chloramphenicol (98%, 49/50). A lower resistance rate was detected for enrofloxacin (18%, 9/50). Only two isolates (4%) showed resistance to ceftazidime and ofloxacin. For amikacin, 46 isolates (92%) were susceptible, 4 isolates (8%) showed intermediate susceptibility, and none was resistant (Table 1). To further characterize the multidrug resistance profiles, isolates were grouped according to the number of antimicrobial classes to which they were resistant (Figure 2). The most prevalent pattern was resistance to six classes, observed in 80% (40/50) of the isolates. Resistance to five classes accounted for 10% (5/50), and resistance to four classes represented 4% (2/50). Only 2% (1/50) of the isolates showed resistance to three classes, and 4% (2/50) were resistant to fewer than three classes. Notably, 96% (48/50) of the isolates were classified as multidrug-resistant (resistant to o3 antimicrobial classes), underscoring the extensive antimicrobial resistance burden among *mcr-1*-positive APEC strains in this region.

**Table 1:** Antimicrobial susceptibility testing results of *mcr-1*-positive strains.

Antibiotics class	Antibiotics name	Number of strains/Number			Percentage of resistance
		Sensitivity	Intermediate	Resistance	
Amides	Chloramphenicol	1	0	49	98.00%
	Florfenicol	2	4	44	88.00%
β-lactams	Amoxicillin	1	2	47	94.00%
	Ceftazidime	47	1	2	4.00%
Quinolones	Enrofloxacin	0	41	9	18.00%
	Ofloxacin	43	5	2	4.00%
Tetracyclines	Tetracycline	0	0	50	100.00%
Sulfonamides	trimethoprim-sulfamethoxazole	0	0	50	100.00%
Aminoglycosides	Amikacin	46	4	0	0.00%
	Kanamycin	1	5	44	88.00%

### 3.3 Identification of antimicrobial resistance genes

**Figure 3:**
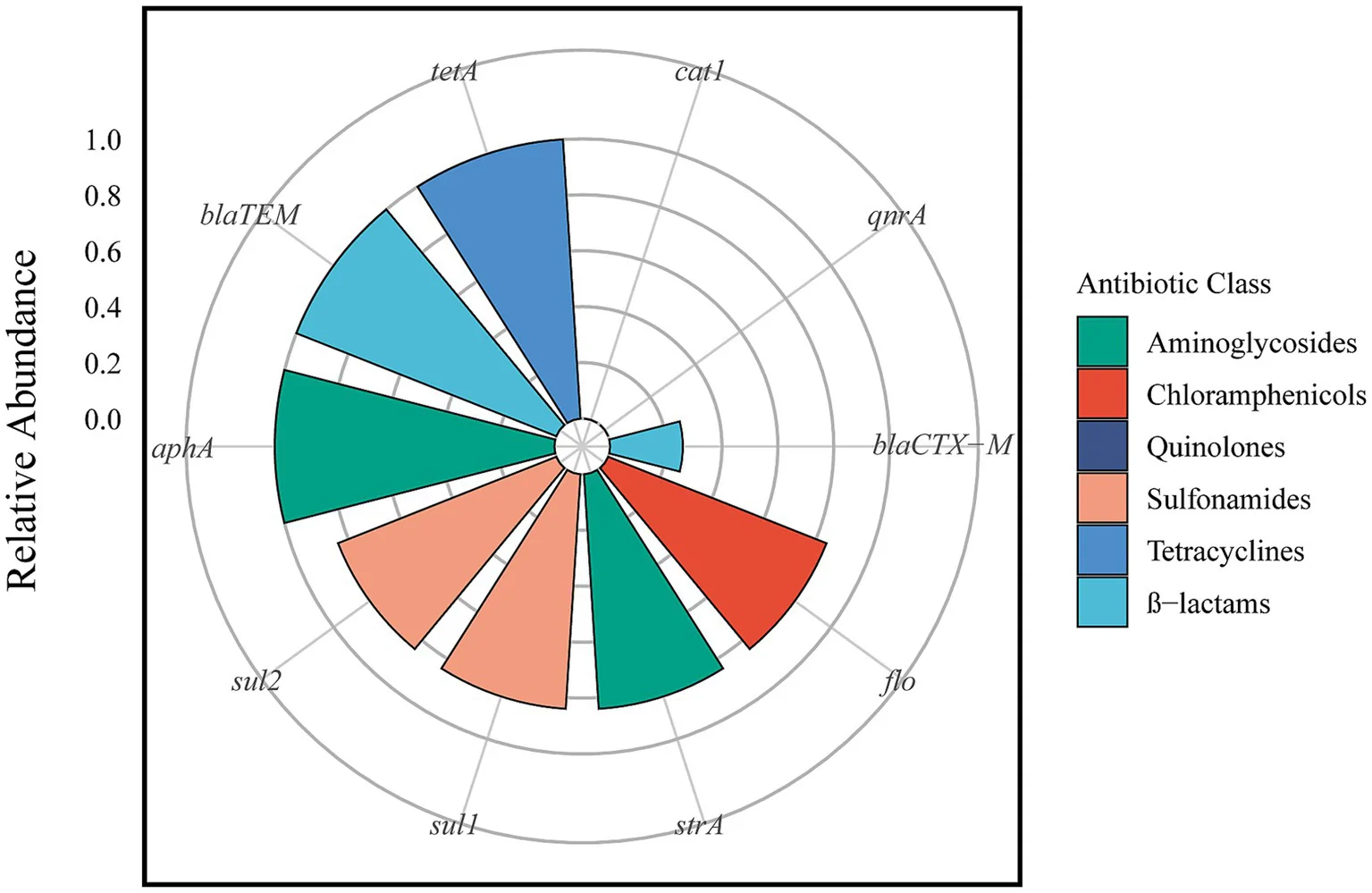
Polar bar chart depicting the distribution of antimicrobial resistance genes among *mcr-1*-positive isolates. The polar bar chart illustrates the proportion of each antimicrobial resistance gene. The outermost ring represents individual genes, arranged counterclockwise in descending order of prevalence: *tetA, blaTEM, aphA, sul2, sul1, strA, floR, blaCTX-M, qnrA*, and *cat1*. Rings from the second outermost to the innermost correspond to proportions of 100, 80, 60, 40, 20, and 0%, respectively.

To analyze antimicrobial resistance genes in the 50 *mcr-*1-positive isolates, PCR assays were performed for each strain. The tetracycline resistance gene *tetA* was detected in all isolates (100%, 50/50). Among amphenicol resistance genes, *cat1* was not detected (0%, 0/50), whereas *floR* was present in 84% (42/50) of the isolates. For *β*-lactam resistance, *blaTEM* was detected in all isolates (100%, 50/50), while *blaCTX-M* was identified in 26% (13/50). The aminoglycoside resistance genes *strA* and *aphA* were detected in 84% (42/50) and 100% (50/50) of the isolates, respectively. The quinolone resistance gene *qnrA* was not detected (0%, 0/50). For sulfonamide resistance, *sul1* and *sul2* were detected in 84% (42/50) and 100% (50/50) of the isolates, respectively (Figure 3).

### 3.4 Distribution of virulence-associated genes

**Figure 4:**
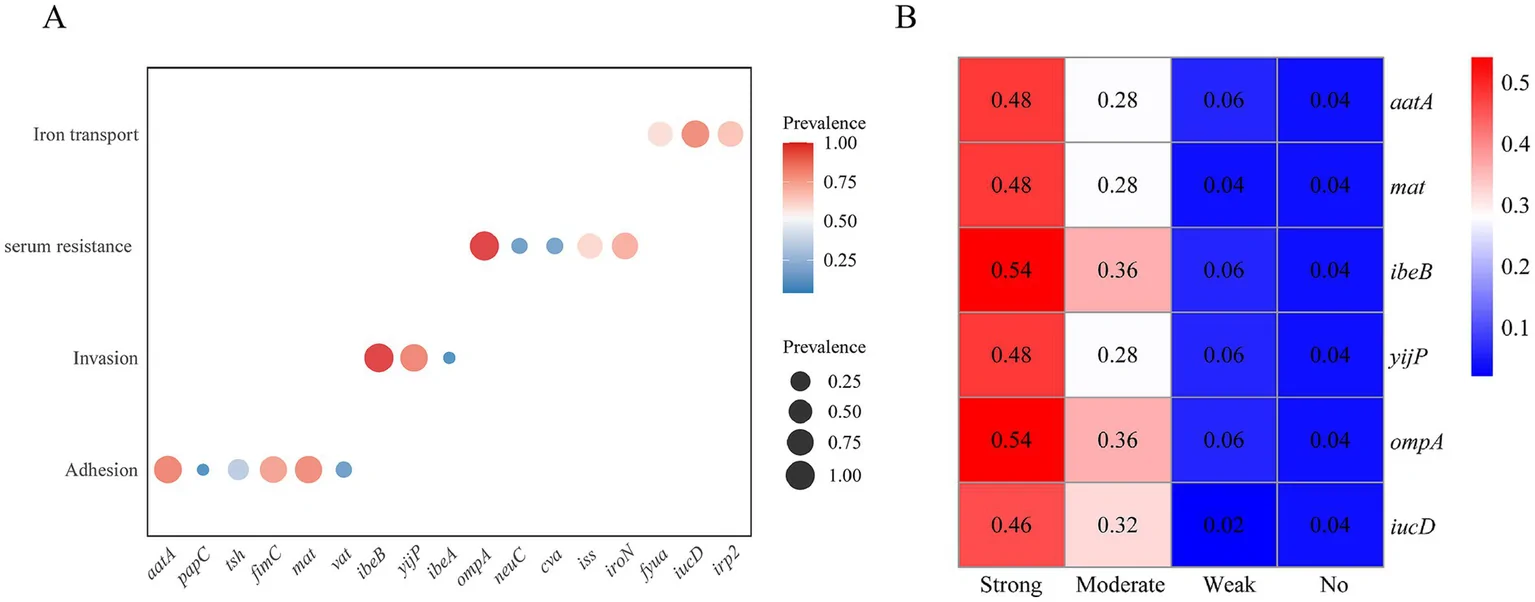
Virulence gene profiles of *mcr-1*-positive isolates. **(A)** A colored bubble plot showing the prevalence of virulence genes among the isolates. The x-axis indicates individual virulence genes, and the y-axis represents functional categories of virulence genes. Bubble size and color intensity reflect the proportion of each gene. **(B)** A heatmap showing the biofilm-forming ability of isolates carrying virulence genes with detection rates above 80%. The x-axis indicates biofilm formation categories, and the y-axis represents virulence gene groups. Color intensity reflects the proportion of each category.

A total of 17 virulence-associated genes were identified among the isolates, and the results are summarized in Figure 4A. Virulence genes with detection rates exceeding 80% included the adhesin-related genes *aatA* (86%, 43/50) and *mat* (84%, 42/50); the invasion-related genes *ibeB* (100%, 50/50) and *yijP* (86%, 43/50); the serum survival–associated gene *ompA* (100%, 50/50); and the iron acquisition gene *iucD* (84%, 42/50). In addition, the adhesin-related gene *fimC* (78%, 39/50); the serum resistance–associated genes *iss* (62%, 31/50) and *iroN* (74%, 37/50); and the iron transport–related genes *fyuA* (60%, 30/50) and *irp2* (68%, 34/50) showed detection rates above 60%. In contrast, relatively low detection rates were observed for *papC* (4%, 2/50), *tsh* (32%, 16/50), *vat* (10%, 5/50), *ibeA* (4%, 2/50), *neuC* (10%, 5/50), and *cva* (12%, 6/50). The distribution of the six virulence genes with detection rates above 80% was further compared among *E. coli* isolates with different biofilm-forming capacities (Figure 4B). The genes *aatA, mat, ibeB, yijP, ompA*, and *iucD* were most frequently detected in strong biofilm-forming isolates, with proportions exceeding 46%. Moderate biofilm-forming isolates showed intermediate detection rates, ranging from 28 to 36%. In contrast, weak or non-biofilm-forming isolates exhibited similarly low detection rates for these six genes, ranging from 2 to 6%. These results indicate that, among *mcr-1*-positive isolates, stronger biofilm-forming ability is associated with a higher likelihood of carrying these virulence genes.

### 3.5 Assessment of biofilm-forming capacity

**Figure 5:**
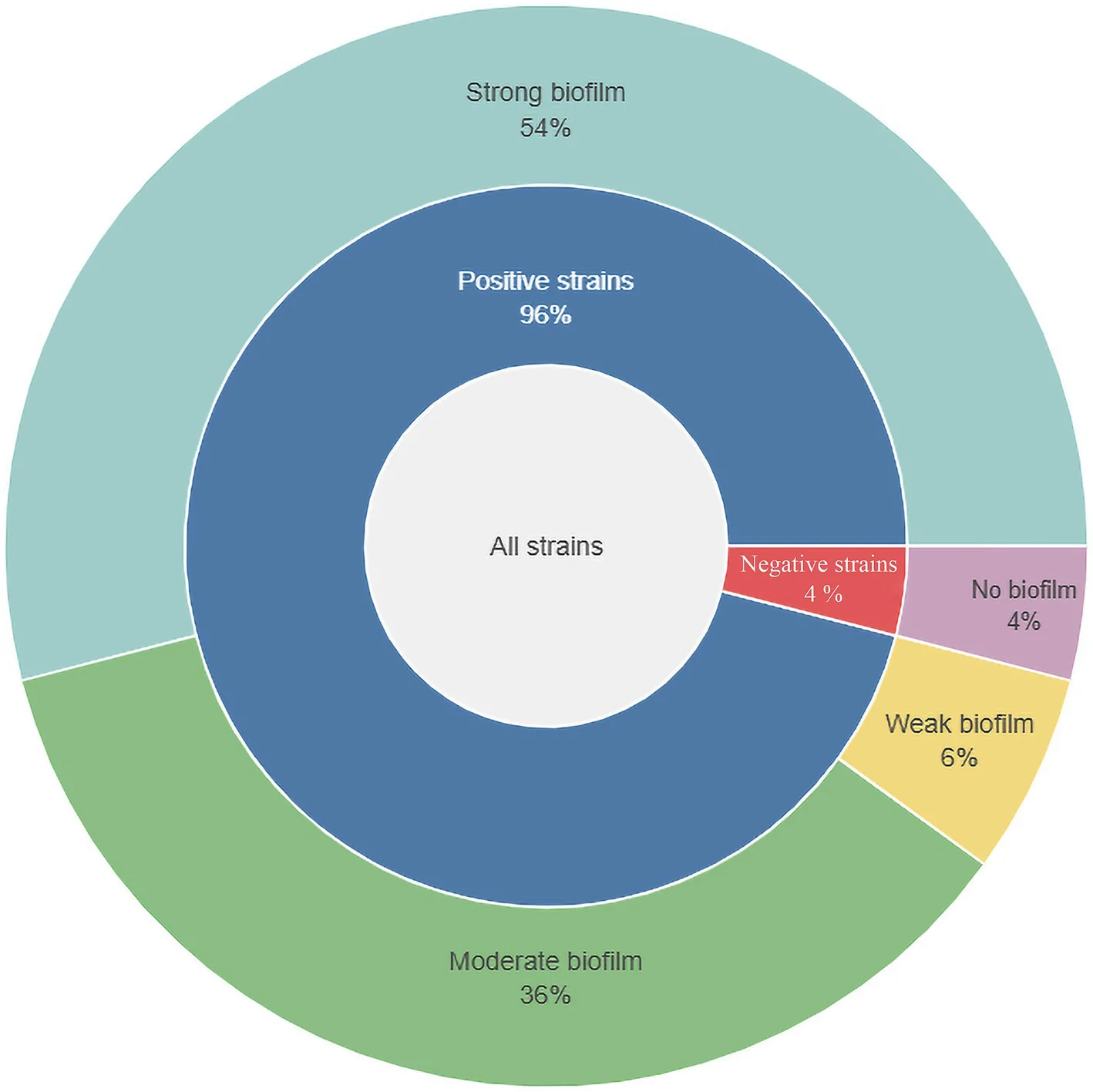
Circular tree diagram showing the distribution of biofilm formation capability among *mcr-1*-positive strains. The outer ring indicates categories and proportions of biofilm-forming capacity. Strong, moderate, weak, and no biofilm formation are shown in light blue, green, orange, and purple, respectively. The inner ring represents the proportions of biofilm-positive and biofilm-negative isolates, shown in dark blue and red, respectively.

The biofilm-forming ability of 50 *mcr-1*-positive APEC isolates was evaluated using the crystal violet staining assay. The results showed that 96% (48/50) of the isolates were capable of forming biofilms. Among them, strong biofilm formation was observed in 54% (27/50) of the isolates, whereas moderate and weak biofilm formation accounted for 36% (18/50) and 6% (3/50), respectively. Only 4% (2/50) of the isolates were classified as non-biofilm formers (Figure 5). These results indicate that *mcr-1*-positive isolates generally exhibit a strong capacity for biofilm formation.

### 3.6 Correlation between biofilm formation and resistance or virulence genes

**Figure 6:**
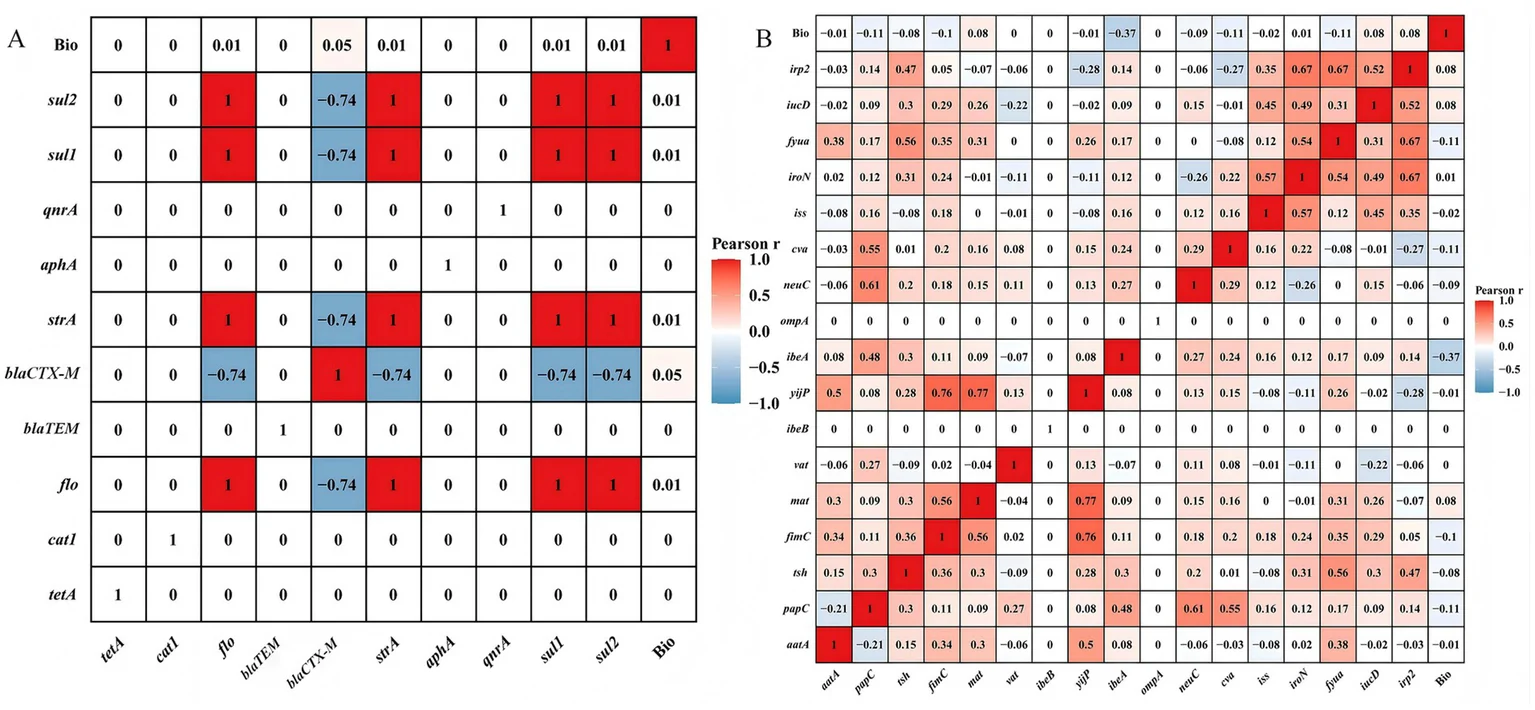
Association of resistance and virulence genes with biofilm-forming capacity. **(A)** Heatmap showing correlations between antimicrobial resistance genes and biofilm-forming capacity. **(B)** Heatmap showing correlations between virulence genes and biofilm-forming capacity. Gene names are shown on both axes, and correlation strength is grouped and indicated by color intensity.

To evaluate the relationship between biofilm formation and resistance or virulence genes, correlation analyses were performed. As shown in Figure 6A, the absolute correlation coefficients (|*r*|, *p* > 0.05) between resistance genes and biofilm formation were all below 0.1, suggesting no correlation. Similarly, virulence-associated genes, in general, showed no association with biofilm formation. However, as shown in Figure 6B, the *ibeA* exhibited a moderate negative correlation with biofilm formation capacity (*r* = −0.37, *p* > 0.05), indicating a potential inhibitory effect on biofilm formation.

## 4 Discussion

Polymyxin antibiotics play a critical role in both human medicine and agriculture. However, expression of the resistance gene *mcr-1* leads to modification of lipid A on the bacterial outer membrane, thereby reducing its affinity for polymyxins (Khondker and Rheinstädter, 2020). Previous studies reported that, among 91 *E. coli* isolates recovered from laying hens, broilers, pullets, and turkeys, phenotypic assays combined with genetic analysis (*mcr-1*) identified a resistance rate of 22% (Stępień-Pyśniak et al., 2025). Of particular concern, this gene can be readily disseminated among different bacterial strains via conjugative horizontal gene transfer, resulting in the widespread and unavoidable emergence of polymyxin resistance, which ultimately poses a serious threat to both animal and human health (Khondker and Rheinstädter, 2020; Rodríguez-Santiago et al., 2021). Therefore, the present study systematically characterized the molecular epidemiological features of 50 *mcr-1*-positive APEC isolates from Shanxi Province, China.

In APEC-associated infections, serogroups O1, O2, O18, O78, and O145 are among the most frequently reported (Tan et al., 2024; Runcharoon et al., 2025). Consistent with previous findings, the common pathogenic serogroups O18 and O78 showed high detection rates in the present study. In contrast, the absence of serogroup O1 may reflect regional or host-related differences. Most APEC strains belong to phylogenetic groups associated with ExPEC. Based on studies of clonal population structure, *E. coli* has been classified into four major phylogenetic groups (A, B1, B2, and D) (Jamali et al., 2024). Ewers et al. (2007) demonstrated, using the *E. coli* reference collection (ECOR), that human ExPEC isolates predominantly belong to phylogroup B2, followed by phylogroup D. Although a considerable proportion of APEC isolates were also assigned to phylogroup B2 (35.1%), a larger proportion (46.1%) belonged to phylogroup A. These observations are consistent with our findings and suggest that the isolates analyzed in this study may be better adapted to environmental niches or avian hosts rather than representing typical human-pathogenic lineages. Notably, 34% of the *mcr-1*-positive isolates in this study could not be assigned to the established *E. coli* phylogenetic group. This may reflect limitations of the PCR-based phylogenetic classification scheme, particularly for APEC strains carrying mobile resistance elements such as *mcr-1*. Further studies employing multiplex PCR approaches are needed to determine whether these isolates belong to additional phylogenetic lineages, including groups C, E, and F (Tohmaz et al., 2022). We acknowledge that the use of the original Clermont triplex method represents a limitation of this study, as it does not distinguish several phylogroups recognised in updated classification schemes. Future studies employing whole-genome sequencing will be necessary to more accurately define the phylogenetic background of these strains.

Sub-therapeutic doses of antibiotics have long been used in poultry feed to prevent and control infections, which has contributed to the increasing prevalence of multidrug-resistant APEC strains (Forgetta et al., 2012). In the present study, all 50 *mcr-1*-positive isolates were identified as multidrug resistant, highlighting the urgent need for effective control strategies against APEC. Azam et al. performed whole-genome sequencing on 75 APEC isolates recovered from colibacillosis-affected broilers in Pakistan and reported that 89.3% of the isolates carried genes predicted to confer resistance to aminoglycosides, tetracyclines, and sulfonamides (Azam et al., 2020). Consistent with these findings, the *mcr-1*-positive isolates in this study exhibited high resistance to most antimicrobial classes, with only a small proportion showing resistance to ceftazidime and ofloxacin, and no resistance to amikacin. Moreover, the observed resistance phenotypes were highly consistent with the presence of resistance genes (*tetA, blaTEM, aphA*, and *sul2*), suggesting that the underlying resistance mechanisms are well defined and relatively stable. Plasmids are extrachromosomal genetic elements and serve as highly efficient vehicles for the horizontal transfer of antimicrobial resistance genes. IncF plasmids, in particular, have been associated with a wide range of resistance determinants targeting clinically important antimicrobials, including quinolones, *β*-lactams, tetracyclines, sulfonamides, chloramphenicol, and aminoglycosides (Yoon et al., 2020). In this study, most resistance genes coexisting with *mcr-1* were plasmid mediated, implying the potential involvement of co-selection or co-dissemination mechanisms.

Wang et al. constructed an *ibeB* mutant of the DE205B strain and demonstrated that *ibeB* is involved in APEC invasion and virulence (Wang et al., 2012). OmpA is a key surface structure that enables bacteria to evade complement-mediated killing during the early stages of infection. *E. coli* K1 can enter neutrophils and macrophages in an OmpA-dependent manner, thereby downregulating the bactericidal functions of these immune cells (Krishnan et al., 2015). In the present study, the *ibeB* and *ompA* genes were detected in all *mcr-1*-positive isolates, indicating that these strains possess strong invasive potential and an enhanced ability to evade host immune defenses.

Biofilms are complex and protective microbial communities that play a critical role in the bacterial life cycle. These structures promote bacterial survival and enhance resistance to environmental stresses, including antibiotic exposure (Wang et al., 2023). In the present study, 96% of the *mcr-1*-positive isolates were capable of forming biofilms, indicating that biofilm formation represents a common and important survival strategy among these strains. We further analysis of the association between biofilm formation and antimicrobial resistance or virulence genes revealed that the majority showed no correlation (|*r*| < 0.1). Interestingly, a moderate negative correlation was observed between the *ibeA* and biofilm formation (*r* = −0.37), which appears to contrast with previous findings in the AAEC189 strain, where *ibeA* was reported to enhance biofilm formation (Wang et al., 2011). This discrepancy may be attributable to substantial differences in the genetic backgrounds of the strains examined. In addition, we found that isolates with strong biofilm-forming capacity exhibited higher carriage rates of virulence genes, suggesting that virulence and environmental adaptability may have co-evolved. Although the correlation analysis revealed no association between biofilm formation and the majority of individual virulence genes, isolates with strong biofilm-forming ability showed a higher cumulative prevalence of multiple virulence factors. These findings suggest that biofilm formation may be associated with pathogenicity at the population level and is likely regulated by multifactorial mechanisms rather than by single-gene effects, warranting further mechanistic investigations.

Several limitations of this study should be acknowledged. First, the isolates analyzed were collected exclusively from Shanxi Province, China, which may limit the generalizability of the findings. Second, whole-genome sequencing was not performed, and therefore novel resistance or virulence genes, as well as detailed plasmid architectures, may have been overlooked. Third, although the resistance phenotypes observed were generally consistent with the detected resistance genes, we acknowledge that other untested resistance mechanisms may also contribute to the phenotypic profiles, as the resistance gene panel investigated was limited. Fourth, we did not examine the genetic context of *mcr-1*, such as plasmid replicon types, transferability, or chromosomal versus plasmid location. These aspects are important for understanding the dissemination of colistin resistance and should be addressed in future studies. In addition, biofilm formation was assessed using an *in vitro* model, which may not fully reflect conditions *in vivo*.

In conclusion, this study demonstrates that *mcr-1*-positive APEC isolates in Shanxi Province, China, exhibit high levels of antimicrobial resistance, possess considerable pathogenic potential, and display a broad capacity for biofilm formation. These findings provide baseline data for understanding the local epidemiology of *mcr-1*-positive APEC in this region. However, as the isolates were collected from a single province, the broader epidemiological significance beyond this area remains to be determined.

By providing baseline data from a key agricultural region in China, our study contributes to the global surveillance of *mcr-1* and enhances our understanding of the epidemiology of *mcr-1-*positive APEC. Future nationwide surveillance, preferably combined with whole-genome analyses, is needed to better assess the distribution and dissemination of these strains. Moreover, strengthened regulation of veterinary antibiotic use is urgently needed to prevent the transmission of resistance genes through the food chain to humans. These findings highlight the need for continued surveillance and coordinated strategies to monitor colistin resistance in poultry production.

## Data Availability

The raw data supporting the conclusions of this article will be made available by the authors, without undue reservation.
