# Supplementary material for: Molecular epidemiology of mcr-1-harboring avian pathogenic Escherichia coli from diseased chickens in Shanxi, China (2021–2024)
Source: Front Microbiol. 2026 Aug 11;17:1883400. doi: 10.3389/fmicb.2026.1883400 (PMC13504654; doi:10.3389/fmicb.2026.1883400)
Supplement: Supplementary file 1 [file Table_1.docx]

Supplementary Table 1. Distribution of sampling sites, E. coli isolation, and detection of **mcr-1**-positive APEC isolates from diseased chickens in Shanxi Province, China (2021–2024)

| Site | Number of sick or dead chickens | Number of *E. coli* isolates | Isolation rate (%) | Number of **mcr-1**-positive APEC isolates | **mcr-1** positivity rate* (%) |
| --- | --- | --- | --- | --- | --- |
| Tai yuan | 66 | 53 | 80.30 | 8 | 15.09 |
| Jin zhong | 98 | 86 | 87.76 | 15 | 17.44 |
| Da tong | 13 | 7 | 53.85 | 1 | 14.29 |
| Lv liang | 46 | 36 | 78.26 | 7 | 19.44 |
| Shuo zhou | 39 | 23 | 58.97 | 2 | 8.70 |
| Chang zhi | 43 | 36 | 83.72 | 6 | 16.67 |
| Lin fen | 51 | 48 | 94.12 | 11 | 22.92 |
| Total | 356 | 289 | 81.18 | 50 | 17.30 |
