# Supplementary material for: Molecular epidemiology of mcr-1-harboring avian pathogenic Escherichia coli from diseased chickens in Shanxi, China (2021–2024)
Source: Front Microbiol. 2026 Aug 11;17:1883400. doi: 10.3389/fmicb.2026.1883400 (PMC13504654; doi:10.3389/fmicb.2026.1883400)
Supplement: Supplementary file 2 [file Table_2.docx]

Supplementary Table 2. O Serotype-Specific Primers used for PCR identification of APEC

| Associated  O serogroup | Primers | Primer sequences (5’-3’) | Length (bp) |
| --- | --- | --- | --- |
| O1 | O1-F | GTGAGCAAA AGTGAAATA AGGAACG | 1098 |
|  | O1-R | CGC TGA TACG AAT ACC ATC CTA C |  |
| O2 | O2-F | TGGCCTTGTTCGATATACTGCGGA | 819 |
|  | O2-R | TCACGAGCTGAGCGAAACTGTTCA |  |
| O18 | O18-F | CGATGTTGAGCGCAAGGTTG | 459 |
|  | O18-R | AGAAGCATTGCGCTGTGGAC |  |
| O78 | O78-F | GGTATGGGTTTGGTGGTA | 992 |
|  | O78-R | AGAATCACAACTCTCGGCA |  |
| O145 | O145-F | CAGGTGAAGGTGGAATGGTTGTC | 296 |
|  | O145-R | TTGAAGTTGAGACCATCCAATAAG |  |
