# Supplementary material for: Molecular epidemiology of mcr-1-harboring avian pathogenic Escherichia coli from diseased chickens in Shanxi, China (2021–2024)
Source: Front Microbiol. 2026 Aug 11;17:1883400. doi: 10.3389/fmicb.2026.1883400 (PMC13504654; doi:10.3389/fmicb.2026.1883400)
Supplement: Supplementary file 3 [file Table_3.docx]

Supplementary Table 3. Primers for detection of phylogenetic clustering genes of *Escherichia coli*

| Gene | Primer | Primer sequences (5’-3’) | Length (bp) |
| --- | --- | --- | --- |
| *yiaA* | yiaA-F | TGAAGTGTCAGGAGACGCTG | 211 |
|  | yiaA-R | ATGGAGAATGCGTTCCTCAAC |  |
| *chuA* | chuA-F | GACGAACCAACGGTCAGGAT | 279 |
|  | chuA-R | TGCCGCCAGTACCAAAGACA |  |
| *TspE4.C 2* | TspE4-F | GAGTAATGTCGGGGCATTCA | 152 |
|  | TspE4-R | CGCGCCAACAAAGTATTACG |  |
