# Supplementary material for: Molecular epidemiology of mcr-1-harboring avian pathogenic Escherichia coli from diseased chickens in Shanxi, China (2021–2024)
Source: Front Microbiol. 2026 Aug 11;17:1883400. doi: 10.3389/fmicb.2026.1883400 (PMC13504654; doi:10.3389/fmicb.2026.1883400)
Supplement: Supplementary file 4 [file Table_4.docx]

Supplementary Table 4. Primers used for detection of common resistance genes of *Escherichia coli*

| Types of resistance genes | Gene | Primer | Sequence (5’-3’) | Product length (bp) |
| --- | --- | --- | --- | --- |
| Tetracyclines | *tetA* | tetA-F | GCTACATCCTGCTTGCCTTC | 210 |
|  |  | tetA-R | CATAGATCGCCGTGAAGAGG |  |
| β-lactams | *blaCTX-M* | CTXM-F | AACCGTCACGCTGTTGTTAG | 766 |
|  |  | CTXM-R | TTGAGGCTGGGTGAAGTAAG |  |
|  | *blaTEM* | TEM-F | GAGTATTCAACATTTTCGT | 857 |
|  |  | TEM-R | ACCAATGCTTAATCAGTGA |  |
| Amides | *cat1* | cat1-F | AGTTGCTCAATGTACCTATAACC | 547 |
|  |  | cat1-R | TTGTAATTCATTAAGCATTCTGCC |  |
|  | *fIoR* | fIoR-F | CGCCGTCATTCCTCACCTTC | 215 |
|  |  | floR-R | GATCACGGGCCACGCTGTGTC |  |
| Sulfonamides | *sul1* | sul1-F | CGGCGTGGGCTACCTGAACG | 433 |
|  |  | sul1-R | GCCGATCGCGTGAAGTTCCG |  |
|  | *sul2* | sul2-F | GCGCTCAAGGCAGATGGCATT | 293 |
|  |  | sul2-R | GCGTTTGATACCGGCACCCGT |  |
| Aminoglycosides | *strA* | strA-F | ATGGTGGACCCTAAAACTCT | 891 |
|  |  | strA-R | CGTCTAGGATCGAGACAAAG |  |
|  | *aphA* | aphA-F | ATGGGCTCGCGATAATGTC | 603 |
|  |  | aphA-R | CTCACCGAGGCAGTTCCAT |  |
| Quinolones | *qnrA* | qnrA-F | AGAGGATTTCTCACGCCAGG | 580 |
|  |  | qnrA-R | TGCCAGGCACAGATCTTGAC |  |
