# Supplementary material for: Molecular epidemiology of mcr-1-harboring avian pathogenic Escherichia coli from diseased chickens in Shanxi, China (2021–2024)
Source: Front Microbiol. 2026 Aug 11;17:1883400. doi: 10.3389/fmicb.2026.1883400 (PMC13504654; doi:10.3389/fmicb.2026.1883400)
Supplement: Supplementary file 5 [file Table_5.docx]

Supplementary Table 5. Primers used for detection of virulence genes of *Escherichia coli*

| Types of virulence genes | Gene | Primer | Sequence (5’-3’) | Product length (bp) |
| --- | --- | --- | --- | --- |
| Adhesion | *aatA* | aatA-F | CTACACTCCGATTCCTCTGG | 713 |
|  |  | aatA-R | CATCCGTTCTGGCACTATTT |  |
|  | *papC* | papC-F | GCACCATTTGTATAATCTGCGCCC | 627 |
|  |  | papC-R | TGAGAGATGATCGTTCCGTCAGGA |  |
|  | *tsh* | tsh-F | GCAGAAAATTCAATTTATCCTTGG | 537 |
|  |  | tsh-R | CTGATAAGCGATGGTGAATTAAC |  |
|  | *fimC* | fimC-F | TATGTTGGCTTTGAAATGGG | 513 |
|  |  | fimC-R | ATCCAGAGCAGCCTGACCTT |  |
|  | *mat* | mat-F | GCAAAGTATTTCTCCCGCATC | 1185 |
|  |  | mat-R | CGGCATCCGCATTATCAAAC |  |
|  | *vat* | vat-F | GTCATTGGACCTGATGGATA | 177 |
|  |  | vat-R | GACTTCATTTGAGCGTGTTG |  |
| **Iron transport** | *fyua* | fyua-F | GGCGGCGTGCGCTTCTCGCA | 209 |
|  |  | fyua-R | CGCAGTAGGCACGATGTTGTA |  |
|  | *irp2* | irp2-F | TGATTTCTGGCGCACCATCT | 794 |
|  |  | irp2-R | GTCATGTTCGGCCAGGATG |  |
|  | *iucD* | iucD-F | TCAGTCATACCGTTGAAAAC | 482 |
|  |  | iucD-R | CAACTCACGATAAATGGTCA |  |
| **Invasion** | *ibeB* | ibeB-F | GCGTGTTGCTGAAGTGAGTG | 585 |
|  |  | ibeB-R | TTGCCAGTTCCAAATGTTCT |  |
|  | *yijP* | yijP-F | ATGATTGTGACTGGCTAACG | 260 |
|  |  | yijP-R | TTGGCAAAGATACCACTTCT |  |
|  | *ibeA* | ibeA-F | GTATGACGGTGGGAACAAGAG | 321 |
|  |  | ibeA-R | TGGCAATAGCAGCGGCAGTC |  |
| **Serum resistance** | *ompA* | ompA-F | ATGATGGTCATCCGTCCCGT | 620 |
|  |  | ompA-R | ATCAGTTCTGCAATAAATGC |  |
|  | *neuC* | neuC-F | AAGAACTTGAAGAGGGAGACT | 774 |
|  |  | neuC-R | CACCGACACTAATGATGACC |  |
|  | *cva* | cva-F | TGCCGTGGTTTAGTCGGATTG | 456 |
|  |  | cva-R | TGCGATTTAAGGCGTTCATG |  |
|  | *iss* | iss-F | TGTCAGGACGGCAGATGAAC | 1491 |
|  |  | iss-R | TTTCCGGACTGCCTCCTTTC |  |
|  | *iroN* | iroN-F | GTATACGATGACGCCGGGAG | 236 |
|  |  | iroN-R | GCCTGTCGCCAGTTATCTGA |  |
